# A ketogenic diet reduces amyloid beta 40 and 42 in a mouse model of Alzheimer's disease

**DOI:** 10.1186/1743-7075-2-28

**Published:** 2005-10-17

**Authors:** Ingrid Van der Auwera, Stefaan Wera, Fred Van Leuven, Samuel T Henderson

**Affiliations:** 1 NV reMYND, Minderbroederstraat 12, 3000 Leuven, Belgium; 2 Experimental Genetics Group, K.U. Leuven – Campus Gasthuisberg O&N-06.602, B-3000 Leuven, Belgium; 3 Accera, Inc., 10901 W 120^th ^Ave., Ste 340, Broomfield, CO, 80021, USA

## Abstract

**Background:**

Alzheimer's disease (AD) is a progressive neurodegenerative disorder that primarily strikes the elderly. Studies in both humans and animal models have linked the consumption of cholesterol and saturated fats with amyloid-β (Aβ) deposition and development of AD. Yet, these studies did not examine high fat diets in combination with reduced carbohydrate intake. Here we tested the effect of a high saturated fat/low carbohydrate diet on a transgenic mouse model of AD.

**Results:**

Starting at three months of age, two groups of female transgenic mice carrying the "London" APP mutation (APP/V717I) were fed either, a standard diet (SD) composed of high carbohydrate/low fat chow, or a ketogenic diet (KD) composed of very low carbohydrate/high saturated fat chow for 43 days. Animals fed the KD exhibited greatly elevated serum ketone body levels, as measured by β-hydroxybutyrate (3.85 ± 2.6 mM), compared to SD fed animals (0.29 ± 0.06 mM). In addition, animals fed the KD lost body weight (SD 22.2 ± 0.6 g vs. KD 17.5 ± 1.4 g, p = 0.0067). In contrast to earlier studies, the brief KD feeding regime significantly reduced total brain Aβ levels by approximately 25%. Despite changes in ketone levels, body weight, and Aβ levels, the KD diet did not alter behavioral measures.

**Conclusion:**

Previous studies have suggested that diets rich in cholesterol and saturated fats increased the deposition of Aβ and the risk of developing AD. Here we demonstrate that a diet rich in saturated fats and low in carbohydrates can actually reduce levels of Aβ. Therefore, dietary strategies aimed at reducing Aβ levels should take into account interactions of dietary components and the metabolic outcomes, in particular, levels of carbohydrates, total calories, and presence of ketone bodies should be considered.

## Background

Alzheimer's disease (AD) is an age-associated neurodegenerative disease that is very common in the US, affecting up to 50% of people between the ages of 75 to 84 years [1]. The number of cases of AD will increase dramatically in the next 50 years due to the aging population of the developed world and will present an increasing medical challenge [2]. Clinically, AD is characterized by progressive impairment in memory and language and is frequently accompanied by behavioral symptoms, such as anxiety and depression. Pathologically, AD is characterized by accumulation of senile plaques, dystrophic neurites, and neurofibrillar tangles. The plaques contain large amounts of the β-amyloid (Aβ) peptide derived from cleavage of the amyloid precursor protein (APP). Mutations in APP that result in increased generation of a particular form of Aβ (Aβ42) have been identified in familial cases of AD and this connection has led to the hypothesis that Aβ is central to the etiology of AD (for review see [3]). However, APP functions as a vesicular transport protein and the etiology of the disease may not be directly related to Aβ, but rather to abnormal cleavage of APP and failure to efficiently move vesicles in the axons [4]. While the precise role of Aβ in AD remains unresolved, it is clear that Aβ serves as a pathological marker for the disease.

The development of AD and the accumulation of Aβ have been linked to dietary factors. Diets rich in saturated fat have been repeatedly implicated in epidemiological studies [5-8], though they have been difficult to reproduce [9]. In addition, several experiments in mouse models seem to confirm the link between lipid rich diets and AD. Using transgenic mouse models of AD several groups have reported that high fat diets or diets with added cholesterol increased levels and deposition of the Aβ peptide [10-14]. However, these studies did not examine the effects of lipid rich diets in combination with low carbohydrate intake.

Diets that contain very low carbohydrate and high fat content are well known to induce the hepatic production of ketone bodies (β-hydroxybutyrate, acetoacetate and acetone) and are often referred to as ketogenic diets (KD). Ketogenic diets in some aspects mimic starvation and were developed for use in humans to treat epilepsy based on the long record of observations that fasting reduces seizures (for review see [15]). The experimental KD is calorie restricted and has fixed composition and is thus different from low carbohydrate diets used for weight loss, which are usually *ad lib *and variable in composition. Despite these differences, low carbohydrate diets may also be effective in preventing seizures and may work through similar mechanisms as a KD [16]. The precise mechanism for the anti-convulsant properties of these diets is still unknown. The low carbohydrate content of both diets induce many metabolic changes that may be protective, such as elevated circulating ketone body levels, increased oxidation of fats, changes in protein metabolism, and changes in gene expression [17,18].

## Results

The present study tested experimentally the effects of a KD composed of extremely low carbohydrate and very high saturated fat content in a transgenic mouse model of AD. The mice express a human APP gene containing the "London" APP mutation (APP/V717I) driven by a *thy-1 *gene promoter. APP/V717I transgenic mice produce significant levels of soluble Aβ in the brain as early as 3 months of age and exhibit extensive plaque deposition by 12–14 months. The animals demonstrate early behavioral deficits and represent a model of early-onset familial AD [19].

### Diet

Sixteen female APP/V717I mice were fed *ad libitum *on a standard diet (SD) comprised of a high carbohydrate/ low fat chow (Muracon-G chow: 35% carbohydrate, 21% protein, 4.5% fat, 39.5% water, fiber, and ash). The predominant fatty acid in Muracon-G is linoleic acid (18:2) and it comprises 1.4% of the chow by weight. At three months of age half the group (8 animals) was switched to a ketogenic diet (KD) comprised of very low carbohydrate/high fat chow while the remaining 8 animals remained on the SD. In both cases the animals had free access to chow at all times and intake was not experimentally limited. For the KD we used Bio-Serv Inc. F3666 chow: 0.76% carbohydrates, 8% protein, 79% fat, 12% water, fiber, and ash. F3666 is a ketogenic chow composed of lard, butter fat, dextrose, casein, fiber, corn oil, mineral mix, and a vitamin mix. F3666 is rich in saturated fats. Greater than 29% of the F3666 chow is composed of saturated fats by weight: 2.4% myristic acid (C14:0), 18.9% palmitic acid (C16:0), and 8.4% stearic (C18:0) (see Table 1). The animals fed the F3666 chow are referred to as the KD group. The mice that remained on the Muracon-G chow are referred to as the SD group.

**Table 1:** Fatty acid profile of F3666 (KD) chow

Fatty acid	gm/kg		gm/kg
C4 Butanoic	4.59	C16 Palmitic	189.03
C6 Hexanoic	3.19	C16:1 cis-9-Hexadecenoic	28.18
C8 Octanoic	2.99	C17 Heptadecanoic	2.38
C10 Decanoic	4.39	C17:1 Heptadecenoic	1.43
C10:1 Decenoic	0.80	C18 Stearic	84.79
C12 Lauric	6.41	C18.1 Oleic	298.34
C12:1 cis-9-Dodecenoic	0.40	C18:2 Linoleic	119.44
C14 Myristic	24.43	C18:3 Linolenic	6.33
C14:1 cis-9-Tetradecenoic	6.14	C20 Eicosanoic	1.18
C15 Pentadecanoic	0.48	C20:1 cis-11-Eicosenoic	3.80

### KD diet and weight loss

**Figure 1:**
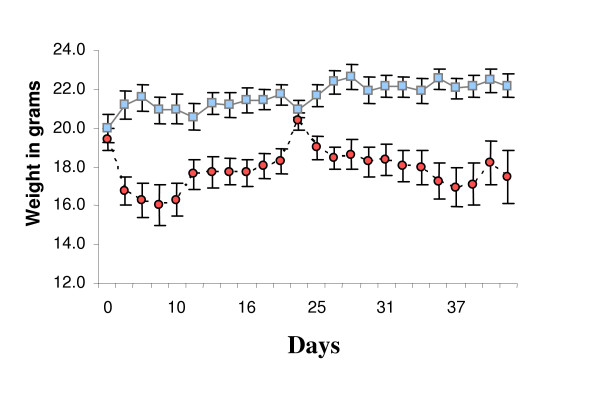
Average weight in grams of each group during the course of the experiment. Blue squares represent standard diet (SD) group. Red circles represent ketogenic diet (KD) group. Error bars represent standard error of the mean. Days signify time in days from start of diet change. Animals on KD lost weight. To mitigate weight loss and improve feeding a small amount of SD chow was mixed with KD chow during the second week and then removed on the third week, see methods.

During the first 7 days many of the animals in the KD group were reluctant to eat the new chow and lost weight (Figure 1). To improve consumption and mitigate weight loss, SD chow was mixed with KD chow at a ratio of 1:3 starting at day 16 until day 20. For the seven days following day 20 the amount of SD chow was reduced to a few crumbs sprinkled over the KD chow. After day 28 the animals were returned to KD chow only. The mixed chow restored body weights of the KD group to approximately the level of the SD group, at about 20 grams (Figure 1). When the animals were fed KD chow exclusively, body weights again dropped, yet tended to stabilize at approximately 18 grams (Figure 1). At the conclusion of the experiment mean weights were significantly different (SD 22.2 ± 0.6 g vs. KD 17.5 ± 1.4 g, p = 0.0067).

### KD diet elevates serum β-hydroxybutyrate levels

**Figure 2:**
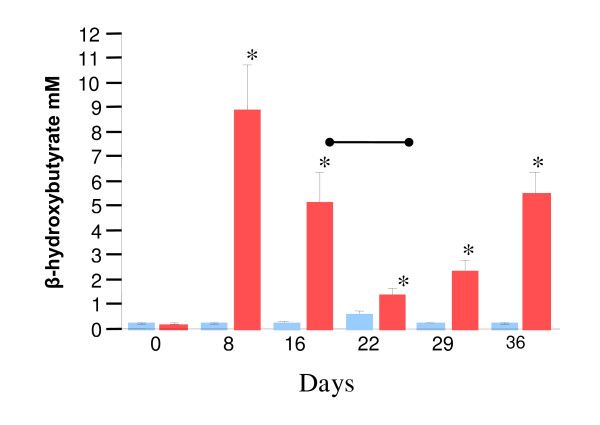
Ketogenic diet induces ketone bodies production. Standard diet (SD) group shown in blue, ketogenic diet (KD) group shown in red, error bars represent standard error of the mean. Serum β-hydroxybutyrate (BHB) levels in mM. * indicates p < 0.05 between KD and SD group. Days signify time in days from start of diet change. Serum β-hydroxybutyrate levels were significantly elevated in KD group at all time points after day 0. Bar indicates period of mixed chow for KD group.

To measure the effectiveness of the chow to induce a ketogenic state, blood samples were taken weekly and examined for levels of β-hydroxybutyrate (BHB). Eight days after switching the chow animals in the KD group had greatly elevated BHB levels compared to the SD group (SD 0.26 ± 0.023 mM vs. KD 8.94 + 1.8 mM, p < 0.0001, Figure 2), possibly due to some animals not eating. As expected, feeding the mixed chow on days 16–28 reduced serum ketone bodies (Figure 2). Yet, at all time points examined after day 0 ketone levels were significantly greater in the KD fed group compared to the SD group (Figure 2). The average serum BHB concentration over the course of the experiment was elevated in the KD group compared to the SD group (SD 0.29 ± 0.06 mM vs. KD 3.85 ± 1.1 mM, p < 0.0078). The elevated ketone levels in the KD group suggested that a metabolic shift toward fat utilization had occurred in these animals.

### Cognitive testing

After 38 days on the diet animals were tested for behavioral deficits using object recognition tests as previously described [20], see methods. Despite the differences in chow, BHB levels, and weight loss, no difference in behavioral measures were detected between the groups (Table 2).

**Table 2:** Recognition Index

Test	SD	KD	p value
RI by time	56.2 ± 5.7	56.7 ± 8.2	0.963
Curiosity by time	10.0 ± 2.7	11.6 ± 6.5	0.803
RI by frequency	49.3 ± 3.6	54.6 ± 9.7	0.628
Curiosity by frequency	14.4 ± 2.3	11.9 ± 3.9	0.530
Mean velocity	3.8 ± 0.4	4.5 ± 0.8	0.447

### Aβ levels

**Figure 3:**
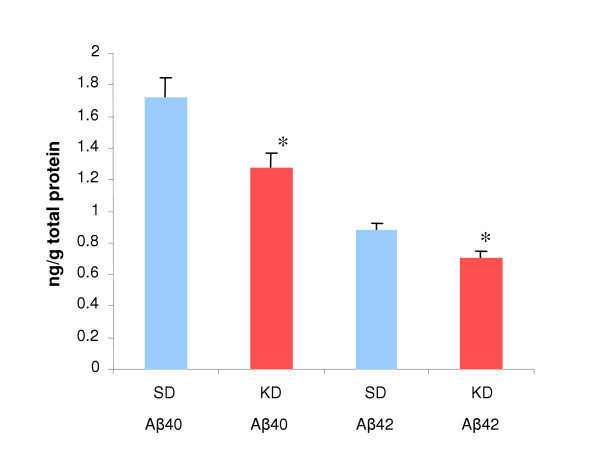
Ketogenic diet reduces Aβ40 and Aβ42. Aβ levels as ng/g of brain tissue. Standard diet (SD) group shown in blue, ketogenic diet (KD) group shown in red, error bars represent standard error of the mean. SD chow Aβ40 1.72 ± 0.12 ng/g vs. KD chow Aβ40 1.28 ± 0.09 ng/g, p= 0.012. SD chow Aβ42 0.88 ± 0.05 ng/g vs. KD chow Aβ42 0.71 ± 0.0.4 ng/g, p = 0.016.

At four months of age APP/V717I mice do not possess Aβ positive plaques and all the Aβ is present in the soluble fraction [19]. Therefore, 43 days after dietary change levels of soluble Aβ in the brain were measured in both groups of animals. Brain homogenate was isolated as previously described [20], see methods. One hemisphere from each animal was analyzed for both Aβ 40 and 42 levels using the Amyloid Aβ40 or Aβ42 ELISA High Sensitivity Kit (The Genetics Company, Zurich, Switzerland). Levels of both soluble Aβ 40 and 42 were found to be significantly lower in the KD fed group (Figure 3). In cases of familial AD excess Aβ42 is produced relative to Aβ40 thereby increasing the Aβ 42/40 ratio. We examined the ratio of Aβ42 to Aβ40 and found no difference between groups (SD 0.51 ± 0.024 vs. KD 0.56 ± 0.026, p= 0.2872), suggesting that the diet did not alter cleavage sites on APP, but instead promoted a general lowering of Aβ species.

Total protein levels were examined to determine if a general decline in brain protein in the KD group could explain the decrease in Aβ levels. However, protein levels, measured as mg/ml of brain homogenate, did not differ between the two groups (SD 0.56 ± 0.035 mg/ml vs. KD 0.51 ± 0.017 mg/ml, p= 0.213). Since most (though not all) of the animals in the KD group lost weight, the animals with the greatest weight loss may have been expected to have the lowest Aβ levels. However, the levels of Aβ40 or Aβ42 did not correlate with weight change across all groups (Aβ40 r = 0.16, p = 0.20; Aβ42 r = 0.05, p = 0.49) or in the KD group alone (Aβ40 r < 0.0001, p = 0.96; Aβ42 r = 0.09, p = 0.62). Both of these measures suggest that the Aβ lowering effect is not the result of a general lowering of protein levels due to weight loss.

A better measure of the effectiveness of the ketogenic diet was serum BHB levels, since all the animals in the KD group exhibited elevated BHB levels. When Aβ40 and 42 levels from all animals were correlated with the average serum BHB over the course of the experiment, a significant correlation was observed (Aβ40 r = 0.42, p = 0.016; Aβ42 r = 0.42, p = 0.016). However, this correlation was most likely driven by the large differences in BHB and Aβ levels between the two groups, since there was no significant correlation between Aβ and BHB levels in the KD group alone (Aβ40 r = 0.083, p = 0.58; Aβ42 r = 0.15, p = 0.46).

## Discussion

This study demonstrated the unexpected result that a brief treatment with a low carbohydrate/high saturated fat diet reduced total Aβ levels in a mouse model of Alzheimer's disease. Previous studies had suggested that diets rich in saturated fats or cholesterol increased both the production and deposition of Aβ in mouse models of AD, leading to the suggestion that diets rich in lipids were a factor in AD [10,12-14]. However, these diets were not low carbohydrate diets. In the high cholesterol diets, cholesterol was added to the diet without reduction in other components [10,14]. In the studies of high fat diets, carbohydrate content was still relatively high. For example, Ho *et al*. used a diet of 60% fat, 20% carbohydrate, 20% protein. This diet was sufficiently high in carbohydrate to cause large increases in body weight in the animals [11].

The interaction of different macronutrients, in particular fats and carbohydrates, is known to influence the metabolic state of the animal. For example, Marsset-Baglieri *et al*. examined if fat in the diet alone was sufficient to shift energy balance toward fat storage. Yet, rats fed *ad libitum *high fat (50%) diets devoid of carbohydrates did not increase energy intake and did not gain in body adiposity, while animals fed high fat diets (30%) in the presence of carbohydrates (56%) increased energy intake and gained in body fat [21]. Such studies support the view that when fat and carbohydrates are consumed simultaneously, the carbohydrates stimulate insulin secretion and thereby promote storage of fat (for recent review see [22]). Therefore, it is important to consider the macronutrient profile of the diet when examining the effects of dietary fat on biological processes.

In the present study, transgenic animals were fed *ad libitum *a very high fat (79%) diet that was practically devoid of carbohydrates (0.76%). The KD resulted in ketone body production, weight loss, and decreased Aβ levels. Hence, the data presented here suggests that it may not be fats in the diet that increases Aβ levels, but perhaps levels of total calories, carbohydrates, or the metabolic state of the animal.

Epidemiological studies in humans have implicated saturated fats in the diet as a risk factor for Alzheimer's disease. For example, Kalmijn *et al*. correlated eating habits with incidence of dementia after a two year follow-up in a large study of 5,386 subjects in Rotterdam, NL. The results from this analysis led the author to suggest that diets rich in saturated fats and cholesterol increased the risk of several types of dementia [5]. However, after a 6 year follow-up of this same population, no correlation between dementia and fat intake could be identified, leading the authors to conclude "High intake of total, saturated, and trans fat and cholesterol and low intake of MUFA, PUFA, n-6 PUFA, and n-3 PUFA were not associated with increased risk of dementia or its subtypes." [9]. More recent studies have also examined the link between dietary fat and cognitive decline. In a study of 2,560 participants ages 65 and older in the Chicago Health and Aging project, fat intake was measured by food questionnaire and correlated with cognitive testing examined after a 3 and 6 year follow- up. This large study found only weak trends between saturated fat and cholesterol intake and cognitive decline [7]. Both the rodent and human studies highlight the complications of trying to link complex environmental factors, such as eating habits or macronutrient intake, with dementia and Alzheimer's disease. In particular, one complicating factor in the human studies is the normal consumption of large amounts of carbohydrates in modern diets.

The present study demonstrates that, contrary to expectations, transgenic mice fed *ab libitum *a very low carbohydrate/high saturated fat diet present lower levels of Aβ after only 43 days of dietary change. The KD group exhibited low levels of both Aβ40 and the more amyloidic Aβ42, suggesting that the KD diet did not change or increase the efficiency of cleavage sites within APP. Instead the data suggests the KD regime either reduced processing of APP or increased degradation of Aβ species. Most of the animals administered the ketogenic diet lost body weight as well as exhibited reduced Aβ levels. However, the reduced Aβ levels may not have been due to a general lowering of protein content. Total brain protein levels did not differ between the groups and Aβ levels did not correlate with weight loss. Interestingly, despite change in diet, weight loss, and Aβ levels, no change in cognitive performance was observed (Table 2). This observation agrees with the general finding that KD diets are not harmful to mice [23]. Also, the finding that reduction in Aβ did not improve cognitive performance may be due to the modest lowering of levels under these conditions and longer treatment may be required.

The KD diet was developed to mimic a starvation response in animals without reducing calories to harmful levels [15]. In this way a KD is similar to caloric restriction (CR) regimes that have been used in many species to alter aging and increase some forms of stress resistance. CR typically reduces calories 30–40% compared to *ad libitum *fed animals and has numerous positive effects on animal health [24]. In the present study we did not attempt to restrict calories in any way and the animals had free access to the ketogenic chow at all times and intake was self limited. However, since the animals were reluctant at first to eat the KD chow and we observed weight loss in the KD group, we cannot rule out the possibility that the Aβ lowering effects were due to CR.

Yet, CR and KD may work through similar mechanisms. KD are well known to reduce insulin signaling and mimic starvation, thereby increasing fatty acid oxidation and promoting a catabolic state [17]. Similarly, CR is well known to reduce serum insulin and IGF levels and much of the benefit of CR may derive from this reduction in insulin/IGF signaling (for review see [25]). For example, decreased insulin/IGF-like signaling inhibits protein synthesis and promotes protein degradation, which may lead to the clearing of degradation-sensitive proteins, such as amyloidic peptides.

Increasing evidence suggests a role for insulin/IGF-1 in regulating APP and modulating Aβ levels. Receptors for both insulin and IGF-1 are highly expressed in brain, especially in hippocampus and cortex, where they may influence learning and memory [26]. Insulin signaling in the brain increases extracellular levels of Aβ by promoting secretion [27] and inhibiting degradation by insulin-degrading enzyme [28]. This view has also gained recent support in humans. Fishel *et al*. demonstrated that induced hyperinsulinemia in healthy elderly subjects elevated both serum and spinal fluid Aβ levels, suggesting insulin plays a role in elevating Aβ, especially under conditions such as type II diabetes [29].

Such an interpretation is consistent with recent studies demonstrating similar Aβ lowering effects of a low carbohydrate, caloric restriction (CR) regime in mice expressing the "Swedish" form of APP (Tg2576 mice). In these animals, lower levels of Aβ 40 and 42 were detected in animals fed 30% less carbohydrates than *ad libitum *fed animals. The Aβ lowering effect may have been due to increased α-secretase and insulin degrading enzyme activity in the CR animals [30].

Alternatively, other physiologic changes may have reduced Aβ levels in this study. The high levels of ketone bodies alone may have contributed to increased protein turnover. Ketone bodies added to cell culture have been shown to lead to increased oxidation of proteins prone to oxidative damage. The presence of damaged protein triggers proteolysis of normally long lived proteins via chaperone-mediated autophagy [31]. Such a mechanism may be at work in animals fed a ketogenic chow and exposed to high levels of ketone bodies. Some support for this model comes from the observation that elevated ketone body levels correlated better with lowering of Aβ species than did weight loss. In addition, ketone bodies may serve as an efficient substrate for neuronal metabolism. Previous studies have shown that acute elevation of ketone bodies may improve cognitive performance in some individuals with mild to moderate AD [32].

## Conclusion

As the population of the developed world ages the incidence of AD is predicted to increase dramatically and will place a tremendous burden on health services [2]. Dietary intervention represents a relatively safe and readily available method to combat AD. Yet, the key dietary links remain unclear. Much of the earlier work has focused on the role of high fat or high cholesterol diets and their contribution to AD. However, evidence suggests that the primary genetic risk factor for late onset AD, the epsilon4 allele of apolipoprotein E, may have been selected against in populations with long historical exposure to agriculture [33]. In addition, foods rich in carbohydrates are relatively recent additions to the human diet and are likely to be more evolutionarily discordant than high fat diets [34]. Therefore, the recent evolutionary switch to high carbohydrate (HC) diets may play an important role in development of AD. HC diets are well known to stimulate insulin signaling and result in a suppression of lipid metabolism [22]. Thus, such diets may lead to inappropriate lipid environments in neurons, mis-cleavage of APP and the resulting inhibition of cellular trafficking, and ultimately increasing the risk of developing AD (for overview see [35]).

## Methods

### Animals

Sixteen APP [V717I] C57Bl × FVB female mice of 3 months of age were used for this study. Mice were housed under a reversed day-night rhythm: 14 hours light/10 hours darkness starting at 7 p.m. in standard metal cages type RVS T2 (area of 540 cm2). The number of mice per cage was limited in accordance with legislation on animal welfare. All mice were genotyped by polymerase chain reaction (PCR) at the age of 3 weeks. Mice were blind randomized and age-matched and had free access to pre-filtered and sterile water (UV-lamp). Mice had free access to either ketogenic (KD) (code F3666, Bio-Serv, Frenchtown, US) or standard (SD) chow (Muracon-G, Trouw Nutrition, Gent). The F3666 chow is a runny paste and was given in special designed liquid food suppliers and was refreshed daily. F3666 is a liquid chow and the animals frequently spilled the chow in the cage. Also, since all the animals in a given group were housed together in a single cage, measuring chow intake per animal was not possible and was not recorded. Due to some problems with weight loss in animals in the KD group, these animals were fed a mixed chow 1(SD):3(KD) starting at day 16 until day 20. From days 21–27 the amount of SD chow was reduced to a few crumbs sprinkled over the KD chow. After day 28 the animals were returned to KD chow only. However, one mouse in the KD group refused food intake and died despite attempts of feeding via gavage. One control animal died during blood draw.

### Blood collection and analysis

Blood was collected from anesthetized mice from either the orbital plexus or via a heart puncture. β-hydroxybutyrate levels were measured spectrophotometrically using the Stanbio liquicolor β-hydroxybutyrate kit (Stanbio Inc., Boerne, Texas).

### Novel object recognition test

The novel object recognition test was performed after 38 days of treatment using the method described by Dewachter *et al*. [20]. Briefly, mice were familiarized for one hour to a Plexiglas open-field box (52 × 52 × 40 cm) with black vertical walls and a translucent floor, dimly illuminated by a lamp placed underneath the box. The next day the animals were placed in the same box and submitted to a 10 minute acquisition trial. During this trial mice were placed individually in the open field in the presence of 2 × object A (orange barrel or green cube, similar sized of ± 4 cm), and the duration (time AA) and the frequency (Freq AA) exploring object A (when the animals snout was directed towards the object at a distance of < 1 cm and the mice were actively sniffing in the direction of the object) was recorded by a computerized system (Ethovision, Noldus information Technology, Wageningen, the Netherlands). During the 10 minute retention trial (second trial) performed 3 hours later, a novel object (object B, green cube or orange barrel) was placed together with the familiar object (object A) into the open field. (Freq A and Freq B and Time A and Time B, respectively). The recognition index (RI), defined as the ratio of the duration in which the novel object was explored over the duration in which both objects were explored (Time B/(Time A + Time B) × 100), was used to measure non-spatial memory. The duration and frequency object A was explored during the acquisition trial (Time AA and Freq AA) was used to measure curiosity.

### Analytical techniques

The mice were anaesthetized with a mixture of Ketalar^® ^(Ketamin), Rompun^® ^(Xylazin 2%) and Atropine (2:1:1) and flushed trans-cardially with physiological serum at 4°C. This was performed to remove blood from the brain vessels, a procedure which has no influence on organ integrity. Blood was collected via a heart puncture and a 1 ml syringe in heparinized Eppendorf tubes. The brain was removed from the cranium and hindbrain and forebrain were separated with a cut in the coronal/frontal plane. The forebrain was divided evenly into left and right hemisphere by using a midline sagittal cut. One hemisphere of each animal was homogenized using a Potter, a glass tube (detergent free, 2 cm^3^) and a mechanical homogenizer (650 rpm). A volume of 6.5 × 1/2 brain weight of freshly prepared 20 mM Tris/HCl buffer (pH 8.5) with Proteinase Inhibitors (1 tablet per 50 ml Tris/HCl buffer, CompleteTM, Roche, Mannheim, Germany) was used as homogenization buffer. The homogenates were collected in Beckman centrifuge tubes TLX and collected on ice prior to centrifugation. Before the samples were centrifuged, 10 % of the sample was used for determination of the total protein concentration of the homogenate whereas 90 % of the samples were centrifuged to process further for biochemistry. The supernatant (soluble fraction containing secreted APP and amyloid peptides) was separated from the pellet by centrifugation (membrane fraction containing membrane-bound APP-fragments). The supernatant was processed further by column chromatography to concentrate the amyloid peptides using small reversed phase columns (C18-Sep-Pack Vac 3cc cartridges, Waters, Massachusetts, MA). Amyloid peptides were eluted with 75% A-TFA and the eluates were collected in 2 ml tubes on ice. Eluates were freeze-dried in a speedvac concentrator (Savant, Farmingdale, NY) overnight and resolved in 240 μl of the sample diluent furnished with the ELISA kits. To quantify the amount of human Aβ40 and human Aβ42 in the soluble fraction of the brain homogenates, commercially available Enzyme-Linked-Immunosorbent-Assay (ELISA) kits were used (h Amyloid Aβ40 or Aβ42 ELISA high sensitive, The Genetics Company, Zurich, Switzerland). Quantification of the Aβ content of the samples was obtained by comparing absorbance to a standard curve made with synthetic Aβ40 or Aβ42. Total protein concentration in the brain homogenate was measured using Bradford solution (Pierce Inc.).

## List of abbreviations

AD, Alzheimer's disease; APP, amyloid precursor protein; Aβ, amyloid beta; SD, standard diet; KD, ketogenic diet; CR, caloric restriction; BHB, β-hydroxybutyrate.

## Competing interests

As co-founder of Accera, Inc., STH holds shares in an organization and may gain or lose financially from the publication of this manuscript. In addition, STH has applied for patents relating to the content of the manuscript and may gain or lose financially from publication of this manuscript.
